# American Otological Society

**Published:** 1888-10

**Authors:** 


### THE AMERICAN OTOLOGICAL SOCIETY.

SEPTEMBER 18.

Professor J. S. PROUT, of Brooklyn, President, in the chair.

Dr. S. O. RICHEY, of Washington, read a paper on "The Primary Physiological Purpose of the Membrana Tympani," which appears elsewhere in this number of this journal.

Professor CHARLES H. BURNET, of Philadelphia, read a paper on "Chronic Purulent Inflammation of the Tympanic Attic." He claimed that the chief difficulty in the diseases of the attic consisted in the inefficient drainage caused by the intricate irregularities of the ossicles being involved in the suppurative process, and the smallness of the opening in the membrana flaccida. Relief was most readily obtained by the use of the tympanic syringe with solutions of peroxide of hydrogen or 5 per cent. carbolic acid, and the enlargement of the perforation when necessary, and possibly in some cases the removal of the ossicles—the malleus and incus.

Dr. SAMUEL SEXTON, of New York, read another paper on "Excision of the Drumhead and Ossicles for the Cure of Chronic Purulent Inflammation of the Attic of the Tympanum."

In most cases of persistent otorrhœa the tympanic attic is the seat of disease, and it is difficult to reach it with remedies. Having some time since been impressed by the surgery of the joints, and reflecting on the resemblance of these cases to those of chronic joint disease, in the chronic muco-periosteal inflammation, caries of the bony walls, and synovitis of the articular surfaces of the ossicles, he had concluded to try the removal of disease structures, thus securing efficient drainage, from which he claims more satisfactory results are obtained.

In reply to a question relative to the danger of wounding the chorda tympani, Dr. Sexton confessed to having had some difficulties in this respect, but added that he thought in many cases calling for the treatment the chorda tympani was already destroyed.

Dr. Gorham Bacon, of New York, read a paper on "A Case of Cerebral Abscess Following Extensive Necrosis of the Temporal Bone. Operation. Death from Secondary Hæmorrhage." The patient was a man of thirty, had had chronic purulent discharge from left ear for two years. Polypi and granulations were removed through the external meatus, and a sinus was found leading upwards from the meatus. Left facial paralysis developed in a short time. An abscess was found above and behind the ear, the opening of which gave some relief. The mastoid region was explored, but no difficulty was found. Dr. Robert F. Weir then made an incision through the temporal muscle, and thrusting his finger through the track toward the base of the skull, relieved a pulsating swelling of about half an ounce of pus. Improvement followed for a short time, but soon became worse, and as a certain form of aphasia had developed, he said "yes, sir" to everything said to him. A counter opening was made in the region of Broca's convolution and a drainage-tube introduced. Secondary hæmorrhage occurred, supposed to have been caused by the drainage-tube pressing on and eroding the middle meningeal artery. Dr. Weir himself suggested that probably the drainage-tube was an error.

Dr. O. D. Pomeroy, of New York, read a paper on "Chronic Suppurative Otitis, with Metastatic Irido-Choroiditis and Abscess on the Side of the Neck Communicating with the Tympanum." The patient was about fifty years of age, and was seen on account of iritis. Soon there was developed pus in anterior chamber. Paracentesis of anterior chamber did not prevent a perforation of the sclera in the upper part. After the eneucleation of the eyeball a stiffness in the neck, which had been previously supposed to be of a rheumatic character, revealed deep fluctuation.

An incision gave vent to several ounces of pus, and pressure caused the pus to appear also in the external meatus. Dr. Pomeroy concluded that the abscess in the ear was the origin of the trouble; that the swelling in the neck was an extension of the difficulty, and that the abscess in the eye was of a metastatic character.

Dr. J. O. Tansley read a paper on "Nasal Difficulties in Ear Disease." Reference was made to the various irregularities and swellings and hypertrophies in the nasal choanæ. The author advocated the removal of such abnormal conditions, either by the snare, saw, forceps, or galvano-cautery. He gave the preference to the cautery in the anterior part of the nose, and used the snare by preference for the removal of the posterior part of the inferior turbinated bones.

Dr. Samuel Theobald, of Baltimore, contributed a paper on the "Employment of Boracic Acid in Solution in the Treatment of Otis Media Suppurativa."

In the ordinary cases of chronic suppurative otitis media, in which there is usually extensive destruction of the drumhead, he uses boracic acid in powder, applying it by means of insufflation, but in acute cases and chronic ones, in which the destruction of the drumhead is not extensive, and in which closure of the perforation may be anticipated, he prefers to use a saturated solution of boracic acid.

The advantages claimed for the solution were that it was never irritating, often gave immediate relief, never blocked up the discharge of pus, and is more readily used at the patient's home.

Dr. GREEN read a paper by Dr. C. J. Blake, of Boston, "On the Influence of the Telephone on the Organ of Hearing"—a very technical paper, which involved a great number of personal observations relative to variations in vibration under different circumstances. The inference was that the persistent use of the telephone may be injurious to persons previously disposed to an affection of the ears.

---

AMERICAN OPHTHALMOLOGICAL SOCIETY.

